# A Novel Mechanism of Macrophage Activation by the Natural Yolkin Polypeptide Complex from Egg Yolk

**DOI:** 10.3390/ijms23063125

**Published:** 2022-03-14

**Authors:** Wioletta Kazana, Dominika Jakubczyk, Katarzyna Pacyga-Prus, Katarzyna Leszczyńska, Sabina Górska, Jakub Siednienko, Józefa Macała, Grażyna Piechowiak, Agnieszka Zabłocka

**Affiliations:** Laboratory of Microbiome Immunobiology, Hirszfeld Institute of Immunology and Experimental Therapy, Polish Academy of Sciences, 12 R. Weigla Str., 53-114 Wrocław, Poland; wioletta.kazana@hirszfeld.pl (W.K.); dominika.jakubczyk@hirszfeld.pl (D.J.); katarzyna.pacyga@hirszfeld.pl (K.P.-P.); katarzyna.leszczynska@hirszfeld.pl (K.L.); sabina.gorska@hirszfeld.pl (S.G.); jakub.siednienko@hirszfeld.pl (J.S.); jozefa.macala@hirszfeld.pl (J.M.); grazyna_piechowiak@wp.pl (G.P.)

**Keywords:** yolkin, macrophages, macrophage polarization, innate immunity, immunomodulators

## Abstract

Ageing is accompanied by the inevitable changes in the function of the immune system. It provides increased susceptibility to chronic infections that have a negative impact on the quality of life of older people. Therefore, rejuvenating the aged immunity has become an important research and therapeutic goal. Yolkin, a polypeptide complex isolated from hen egg yolks, possesses immunoregulatory and neuroprotective activity. Considering that macrophages play a key role in pathogen recognition and antigen presentation, we evaluated the impact of yolkin on the phenotype and function of mouse bone marrow-derived macrophages of the BMDM cell line. We determined yolkin bioavailability and the surface co-expression of CD80/CD86 using flow cytometry and IL-6, IL-10, TGF-β and iNOS mRNA expression via real-time PCR. Additionally, the impact of yolkin on the regulation of cytokine expression by MAPK and PI3K/Akt kinases was determined. The stimulation of cells with yolkin induced significant changes in cell morphology and an increase in CD80/CD86 expression. Using pharmaceutical inhibitors of ERK, JNK and PI3K/Akt, we have shown that yolkin is able to activate these kinases to control cytokine mRNA expression. Our results suggest that yolkin is a good regulator of macrophage activity, priming mainly the M1 phenotype. Therefore, it is believed that yolkin possesses significant therapeutic potential and represents a promising possibility for the development of novel immunomodulatory medicine.

## 1. Introduction

Egg yolk is rich in nutrients and preservative substances with antimicrobial and immunoregulatory activities due to its original role as an embryonic chamber [1,2]. The precursor of major proteins in the egg yolk is vitellogenin, which, during egg formation, is enzymatically cleaved into fragments situated in the yolk plasma or granules [3]. It was shown by Polanowski et al. [4,5] that immunoglobulin Y from hen egg yolks is accompanied by a complex of peptides, named yolkin, possessing immunoregulatory properties.

Yolkin is a heterogeneous set of several peptides with an apparent molecular weight of 1 to about 35 kDa. In this mixture, polypeptides with a molecular weight from 16 to 23 kDa are the most abundant. It has been shown that purified yolkin constituents are homologous with some fragments of the *C-*terminal domain of vitellogenin II. The fractions of MW lower than 12 kDa are free of carbohydrates and their amino acid sequences respond to the sequence of the vitellogenin II, starting from the position 1732aa. Additionally, fractions of MW higher than 16 kDa are glycoproteins corresponding also to the amino acid sequence of the vitellogenin II starting at the position 1572aa [4].

Yolkin possesses immunoregulatory activity and stimulates human whole blood [4,5,6] and mouse macrophages of the BMDM cell line [7] to produce and release inflammatory factors, such as interferons α/β (IFNs α/β), interleukin 1 beta (IL-1β), interleukin 6 (IL-6), tumor necrosis factor alpha (TNFα), nitric oxide (NO) and anti-inflammatory interleukin 10 (IL-10). The neuroprotective and pro-cognitive activity of yolkin has also been determined. Yolkin has moderated ageing symptoms and supported learning functions in both young and old rats [8]. Furthermore, it was found that the peptide complex also has antioxidant activity, which is fundamental for protection against oxidative damage and stimulates neuron-like PC12 cells and human whole blood to secrete the mature form of brain-derived neurotrophic factor (BDNF) [6,9]. These results suggest that the yolkin polypeptide complex may have a positive therapeutic effect both in immunodeficiency and in human neurodegenerative diseases, such as Alzheimer’s disease.

Macrophages are key immune cells involved in the immune response to pathogens, tumors, lifestyle-associated diseases and neurodegenerative disorders [10]. They are highly plastic cells and functionally adapt to signals produced in the local microenvironment [11]. Macrophages play a crucial role in the elimination of pathogens via the production of reactive oxygen and nitrogen species (RONS) and via active phagocytosis. They also play a key role in maintaining homeostasis as they can remove deleterious senescent cells that increase in number during ageing [12]. Macrophages release a wide range of inflammatory factors, including cytokines and chemokines that are crucial to the induction of the inflammatory process [13]. Additionally, macrophages, with their ability to present antigen and activate T and B cells, play an important role in the initiation and propagation of the adaptive immune response [11].

Macrophages are divided into a few subsets based on their need for cytokines to differentiate, function and display distinct phenotypes [13]. These cells have been generally classified into two main subsets, termed M1 and M2 cells [14]. Classical, early activation is induced by interferon gamma (IFN-γ) and lipopolysaccharide (LPS) and produces cells with an M1 phenotype. Macrophages exhibiting the M1 phenotype express a variety of cell surface receptors to help them recognize invading microorganisms. They release nitric oxide (NO) and pro-inflammatory cytokines, such as TNFα, interleukin 12 (IL-12), IL-1β and IL-6, and play an important role in the inflammatory and bactericidal response [15]. However, macrophages can also be activated later in response leading to an anti-inflammatory or regenerative phenotype M2. These regenerative macrophages secrete cytokines such as IL-10, interleukin 13 (IL-13), and transforming growth factor β1 (TGF-β1). M2 cells participate in Th2 response and tissue repair and have immunoregulatory functions [16,17].

Ageing is accompanied by a remodeling of the immune system. The observed decrease in immune efficacy provides increased vulnerability to infectious diseases and increased susceptibility to age-related inflammatory diseases. Age-related changes in the immune system lead to a significant reduction in the ability to trigger an effective innate immune response, which is crucial in protecting against infection [18]. It was shown that macrophages, derived from aged humans and mice, demonstrated a widespread number of functional defects with age. These included impaired phagocytosis, chemotaxis and wound repair, the impaired ability to respond efficiently to new antigens and increased incidence of autoimmunity [10,18,19,20]. The recognition of pathogens by Toll-like receptors (TLR) culminates in the secretion of type I interferons, cytokines or NO, which are responsible for the coordination of the innate immune response. Ageing alters cytokine secretion by macrophages in response to TLR stimulation. It has been shown that the expression of proinflammatory IL-1β and IL-6 were reduced, and the expression of IL-10 was increased in macrophages from old mice. It was suggested that the reduced expression of TLR4 is the cause of the observed age-related downregulation in TLR signaling [21]. It was also indicated that the underlying mechanism is based on the impaired intracellular signaling, leading to a reduction in LPS-induced phosphorylation of the mitogen-activated protein kinases (MAPKs) and the decreased activation of nuclear factor kappa-light-chain-enhancer of activated B cells (NF-kB) [22].

A therapeutic intervention to regulate the aging of the immune system would provide opportunities for the improvement of age-related morbidities and would have a major impact on the health status of older people. Additionally, understanding the mechanisms of age-related disorders in immune regulation is important to generate more efficient therapeutic strategies for immune rejuvenation. One possibility is the use of biologically active immunoregulators capable of restoring ageing-reduced immunity. One of them is yolkin, a polypeptide complex isolated from hen egg yolks. In our previous work, we determined the molecular mechanism of immunoregulatory and antiviral activity of the yolkin complex in mouse bone marrow-derived macrophages (BMDM) [7]. In the present study, we investigated the impact of yolkin on the classical activation of BMDM cells, its polarization, proliferation and regulation by extracellular signal-regulated kinases (ERKs), c-Jun N-terminal kinases (JNKs) and phosphoinositide 3-kinase/protein kinase B (PI3K/Akt) in cytokine mRNA expression.

## 2. Results

### 2.1. Yolkin Uptake by Bone Marrow-Derived Macrophages (BMDM) Cells Is a Time-Dependent Process

**Figure 1:**
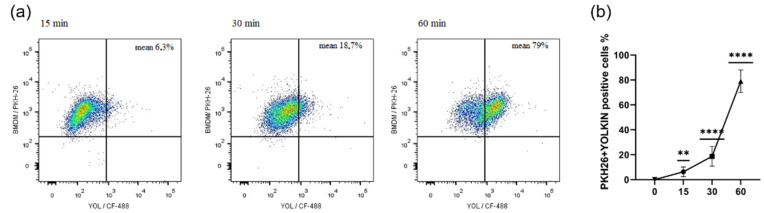
Analysis of yolkin uptake by BMDM cells over time. The cell membrane was stained with red fluorescent cell linker PKH26 prior to seeding on the tissue culture plate (3 × 10^5^/mL). Next, the cells were stimulated with labelled yolkin (100 µg/mL) for 15, 30 and 60 min and then analyzed using FACS. The quantity (%) of double-positive cells (PKH26 + YOLKIN) over time was measured. The results represent three independent experiments with representative flow cytometry dot plots (**a**) and a graph of changes over time, presented as median with SD (**b**). The post-measured analysis was performed using FlowJo VX.07 software, and a one-sample *t*-test was used to examine the mean differences between actual mean and theoretical mean (0), all with ** *p* ≤ 0.005, and **** *p* ≤ 0.0001.

It was shown that yolkin uptake is a dynamic process that depends on the duration of protein exposure (Figure 1). The mean values for yolkin uptake were: 6.3% for 15 min of stimulation (*p* ≤ 0.0024), 18.7% for 30 min (*p* ≤ 0.0001) and 79% for 60 min (*p* ≤ 0.0001).

### 2.2. Yolkin Downregulated BMDM Cells Proliferation

**Figure 2:**
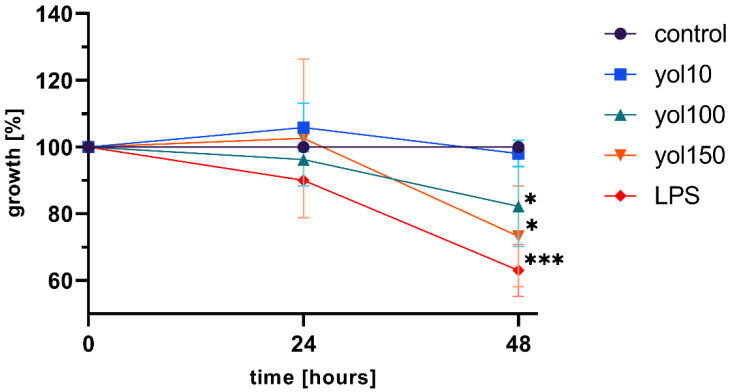
Effect of yolkin on viability and proliferation of bone marrow-derived macrophages (BMDM). The cells (1 × 10^5^/mL) were seeded in 96-well plates in DMEM + 10% FBS and incubated overnight at 37 °C. Next, the cells were exposed to yolkin (10, 100 and 150 µg/mL) for 24 and 48 h. The cell proliferation activity of yolkin was assessed with an MTT assay. Non-stimulated cells were used as a negative control. The results represent three to five independent experiments and data are presented as median ± min–max. A one-sample *t*-test was used to examine the mean differences between samples and control (100). * *p* ≤ 0.05, *** *p* ≤ 0.0001.

We assessed the pro-survival and proliferative effect of yolkin on BMDM cells that were incubated in 96-well plates and maintained in DMEM containing 10% FBS overnight. Then, cells were exposed to three doses of yolkin 10, 100 and 150 μg/mL for 24 and 48 h. The cell growth was measured using an MTT assay. The results are depicted in Figure 2. Yolkin treatment did not cause significant growth inhibition at 10 μg/mL. However, it was shown that the yolkin polypeptide complex significantly downregulated BMDM cell proliferation at doses of 100 and 150 μg/mL after 48 h of stimulation.

### 2.3. Yolkin Induced BMDM Cells Polarization towards M1-like Phenotypes

#### 2.3.1. Morphological Changes

**Figure 3:**
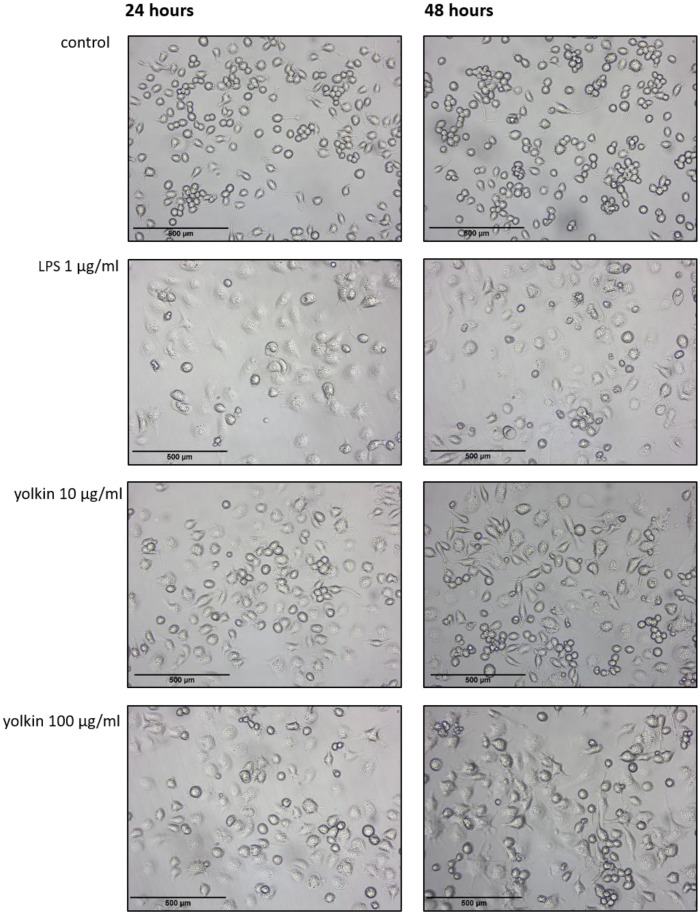
Morphology of yolkin- or LPS-stimulated BMDM cells. Stimulation of BMDM cells for 24 or 48 h with yolkin (10 and 100 µg/mL) or LPS (1 µg/mL) induced morphological changes that were visible under a light microscope with 4× lenses. The cells in the control group showed a round-shaped morphology, which changed after exposure to LPS or yolkin, where more spindle-shaped cells were presented. The cells were examined via light microscopy (Leica DM FRE2, bright field). One representative experiment is shown in this figure. Scale bars represent 500 microns.

Characteristic changes in BMDM cell morphology in response to the examined yolkin were observed under a light microscope with 4× lenses (Figure 3). The majority of BMDM cells treated with yolkin appeared to be more elongated and spindle-shaped than untreated control cells, where the round-shaped cells predominated. Additionally, morphological changes in BMDM influenced by yolkin were comparable to those observed after LPS treatment.

#### 2.3.2. Yolkin Increases the Expression of CD80/CD86 Markers on the Surface of M1 Cells

**Figure 4:**
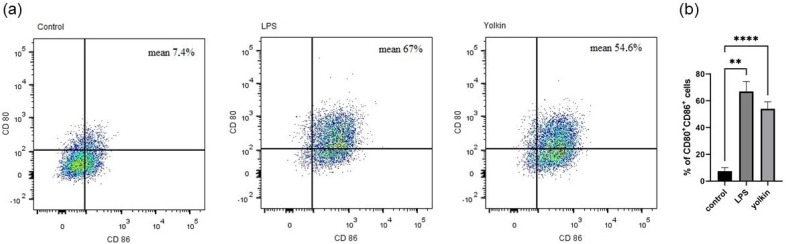
Analysis of co-expression of CD80 and CD86 on BMDM cells. The cells (3 × 10^5^/mL) were stimulated with yolkin (100µg/mL) or LPS (1 µg/mL) for 24 h and then stained with fluorescent CD80 (1:100) and CD86 (1:100) mouse antibodies. The cells were analyzed using FACS. The results represent three independent experiments with representative flow cytometry dot plots (**a**) and a graph summarizing the changes, presented as median with SD (**b**). The cells stimulated with yolkin or LPS were compared with the non-stimulated control cells. The post-measurement analysis was performed using FlowJo VX.07 software and data were analyzed with a one-way ANOVA (Brown–Forsythe and Welch’s test). ** *p* ≤ 0.005; **** *p* ≤ 0.0001.

Macrophage polarization is associated with changes in surface marker expression, participating in their specialized function. It was shown that the LPS-induced macrophage phenotype is characterized by the high surface co-expression of costimulatory molecules CD80 and CD86 (mean 67% for LPS vs. 7.4% for untreated control), which is necessary for the effective presentation of antigens to T cells. The cell surface marker expression of CD80/CD86 on BMDM cells was also significantly enhanced after 24 h of incubation after yolkin was applied to the cells at a dose of 100 μg/mL (mean 54.6% for yolkin vs. 7.4% for untreated control) (Figure 4). These results provide evidence of BMDM polarization towards the M1 phenotype in response to yolkin treatment.

#### 2.3.3. Yolkin Upregulated Pro- and Anti-Inflammatory Cytokines and iNOS mRNA Expression in BMDM Cells

It was shown previously by Kazana et al. [7] that yolkin significantly upregulates mRNA expression and the production of IFNβ1 and TNFα.

**Figure 5:**
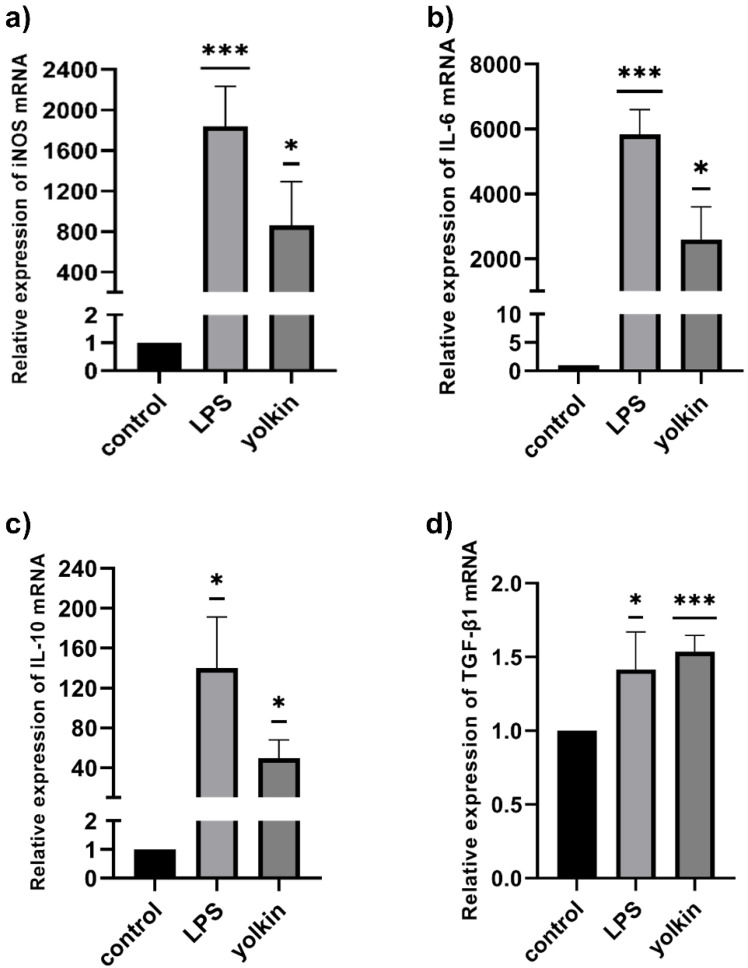
The impact of yolkin on the regulation of iNOS (**a**), IL-6 (**b**), IL-10 (**c**) and TGFβ (**d**) mRNA expression. BMDM cells (1 × 10^6^/mL) were stimulated with yolkin (100 μg/mL) or LPS (1 μg/mL). After 4 h of stimulation, total RNA was isolated from the cells and the expression of iNOS (**a**), IL-6 (**b**), IL-10 (**c**) and TGFβ (**d**) mRNA was determined using real-time PCR tests. Experiments were repeated at least three times and data are presented in relative expression units, where *Actb* was used to normalize all samples. Control cells were assigned an arbitrary value of 1. A one-sample *t*-test was used to examine the mean differences between samples and control; * *p* ≤ 0.05; *** *p* ≤ 0.001 vs. control.

**Figure 6:**
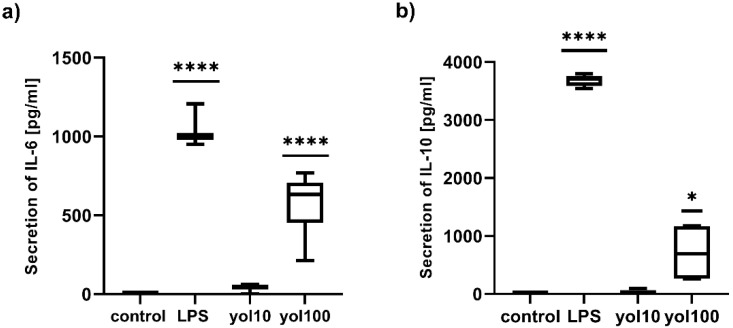
The impact of yolkin on IL-6 (**a**) and IL-10 (**b**) production by BMDM cells. BMDM cells (1 × 10^6^/mL) were stimulated with yolkin (10 and 100 ug/mL) or LPS (1 μg/mL). After 24 h of stimulation, supernatants were collected, and the level of cytokines was determined using ELISA. Results are presented as mean with min–max (*n* = 3–4). A Kruskal–Wallis test was used to examine the mean differences between samples and control * *p* ≤ 0.05; **** *p* ≤ 0.00001; ns (not significant) vs. control.

In the present study, the effect of yolkin on the mRNA expression of IL-6, IL-10, TGFβ and iNOS was determined. Treatment with yolkin induced an 860-fold change in the transcription of iNOS (Figure 5a) and a 2595-fold change in the transcription of IL-6 (Figure 5b) when compared to the control level. At the same time, a 49-fold increase in anti-inflammatory IL-10 mRNA expression (Figure 5c) and a 1.53-fold increase in the transcription of TGFβ (Figure 5d) were shown. The stimulation of BMDM cells with different doses of yolkin (10 and 100 µg/mL) resulted also in the secretion of high amounts of IFNβ1, TNFα and NO, as was presented previously [7], and additionally IL-6 (Figure 6a) and IL-10 (Figure 6b). Additionally, no detectable level of TGFβ was observed in cell culture supernatants after yolkin stimulation (data not shown). These results indicate that the yolkin activates BMDM cells more similarly to the classical M1 profile.

#### 2.3.4. Yolkin Upregulated Cytokine mRNA Expression through the Activation of MAPK and PI3K/Akt Kinases

**Figure 7:**
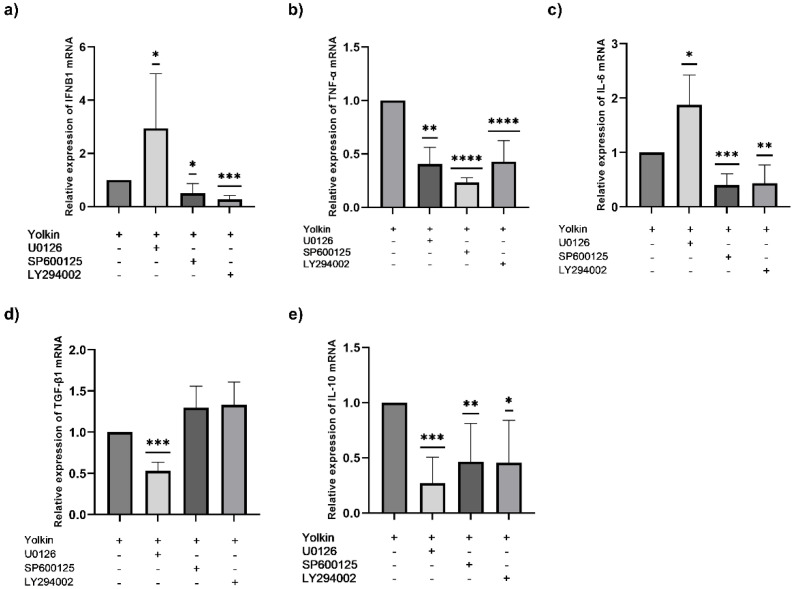
MAPK and PI3K/Akt-dependent regulation of cytokine expression after yolkin treatment of BMDM cells. BMDM cells (1 × 10^6^/mL) were pre-incubated for 1 h with selective kinase inhibitors: 20 μM U0126 (ERK1/2), 25 μM SP600125 (JNK) or 20 μM LY294002 (PI3K/Akt). Next, the cells were stimulated with yolkin (100 μg/mL) for 4 h. After stimulation, total RNA was isolated from the cells, and the expression of IFNβ1 (**a**), TNF-α (**b**), IL-6 (**c**), TGFβ (**d**) and IL-10 (**e**) mRNA was determined via real-time PCR. Experiments were repeated at least three times and data are presented in relative expression units, where *Actb* was used to normalize all samples. Yolkin-treated cells were assigned an arbitrary value of 1. Results are presented as mean ± SD. A one-sample *t*-test was used to examine the mean differences between yolkin-treated samples and yolkin with inhibitor-treated groups; * *p* ≤ 0.05, ** *p* ≤ 0.001, *** *p* ≤ 0.0001; **** *p* ≤ 0.00001; vs. yolkin.

The results mentioned above show that yolkin could promote macrophage polarization and induce the mRNA expression of inflammatory mediators such as IL-6, TGFβ, IL-10, and iNOS. Previously published data by Kazana et al. (2020) showed that yolkin also upregulates IFNβ1 and TNF-α mRNA, and that the MAPK signaling pathway is involved in macrophage activation induced in response to yolkin. Therefore, in this study, we deciphered the impact of yolkin on the MAPK- and PI3K/Akt-dependent upregulation of cytokine mRNA expression using appropriate inhibitors: MEK1/2 inhibitor (U0126), JNK1/2/3 inhibitor (SP600125), and additionally PI3K/Akt inhibitor (LY294002). BMDM cells were pre-incubated for 1 h with selected kinase inhibitors and were next incubated with yolkin (100 µg/mL) for 4 h. The mRNA levels of IFNβ1 (Figure 7a), TNFα (Figure 7b), IL-6 (Figure 7c), TGFβ (Figure 7d) and IL-10 (Figure 7e) were determined via RT-qPCR. The obtained results showed that the pretreatment of BMDM cells with U0126, SP600125 or LY294002 inhibitors significantly reduced the yolkin-dependent expression of TNFα and IL-10, which suggests that both MAPK and PI3K/Akt pathways control TNFα and IL-10 mRNA expression. In the case of IFNβ1 and IL-6, the pretreatment of BMDM cells with SP600125 or LY294002 significantly reduced the yolkin-dependent expression of these cytokines, which indicates that JNK and PI3K/Akt kinases participate in the activation of type I interferons and interleukin 6. However, a significant increase in both IFNβ1 (Figure 7a) and IL-6 (Figure 7c) mRNA was observed when BMDM cells were treated with MEK1/2 kinase inhibitor-U0126. In the case of TGFβ, the pretreatment of BMDM cells only with MEK1/2 kinase inhibitor-U0126 significantly reduced yolkin-dependent TGFβ expression (Figure 7d), which was very weak compared to the other cytokines expression. Additionally, the involvement of PI3K/Akt and JNK kinases in the regulation of the expression of this cytokine was not demonstrated.

#### 2.3.5. Yolkin Was Recognized by TLR2 and TLR4 Receptors

**Figure 8:**
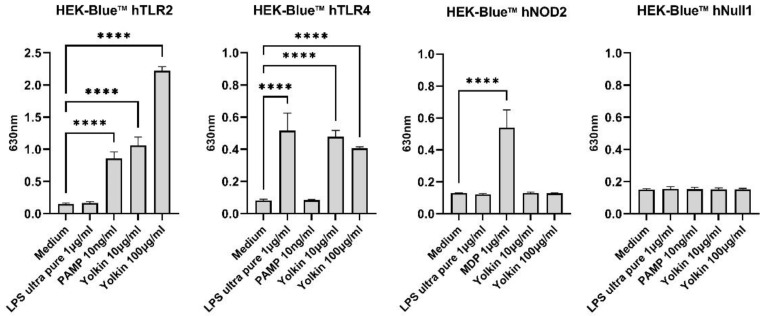
Recognition of yolkin samples by innate immune receptors. HEK Blue^TM^ cells were seeded on the plate and stimulated with yolkin samples in 3 repetitions and at concentrations of 10 µg/mL and 100 µg/mL according to the manufacturer’s instruction. To analyze yolkin recognition over time, a set of absorbance measurements was performed at 610, 630 and 650 nm. Then, 630 nm was selected as the representative. LPS was used as a positive control for TLR4 cells and negative for TLR2 and NOD2 cell lines. A positive control for TLR2 recognition was PAMP and for the NOD2 receptor it was MDP. All of the obtained results were statistically significant. A one-way ANOVA with Dunnett’s multiple comparison test was used to compare treated cells with non-treated control (medium). **** *p* ≤ 0.0001.

HEK-Blue^TM^ cells are engineered human embryonic kidney 293 (HEK 293) cells that, due to transfection, are able to express innate immune receptors from the Toll-like (TLR) and nucleotide oligomerization domain (NOD) receptor families. The stimulation of those cells with receptor ligands results in a colorimetric reaction that can be observed over time, which makes them a perfect candidate for recognition pathway studies. In our research, HEK-Blue^TM^ hTLR2/hTLR4/NOD2 and hNull1 cells were used for stimulation with yolkin sample at the concentration of 10 µg/mL and 100 µg/mL (Figure 8). The results showed that yolkin was strongly recognized by the TLR2 receptor in a dose-dependent manner. We also observed a weaker activation of the TLR4 receptor; however, the level of the response was not dose-dependent. Moreover, NOD2 did not recognize yolkin samples. Null cells that lack analyzed receptors were used as negative controls for transfected cell lines.

## 3. Discussion

Ageing is accompanied by the inevitable changes in the function of the immune system. It provides increased susceptibility to chronic infections, resulting in a negative impact on the quality of life of older people. Therefore, rejuvenating the ageing immune system and maintaining the good health of older people has become an important research and therapeutic goal [23,24]. One of the possibilities is to use naturally occurring substances that are safe, bioavailable and possess the ability to regulate the activity of the immunologically competent cells. As an example, the yolkin polypeptide complex isolated from hen egg yolks seems to be very promising. However, it is necessary to examine its biological mechanism of action in detail.

In our previous study, we revealed that the yolkin polypeptide complex isolated from hen egg yolks is a good modulator of the immune response. It was shown that yolkin activated the macrophages of BMDM cell line to produce and release innate immunity mediators, such as TNFα, type I Interferons and NO, which are important in the regulation of the immune response against pathogens or cancer cells. Additionally, yolkin modulated the activity of the JNK and ERK1/2 kinases that control the expression of the above-mentioned pro-inflammatory mediators. It was also shown that NO, TNFα and type I IFNs, produced by BMDM cells in response to yolkin, triggered antiviral activity [7]. In the present studies, we extended those earlier observations of Kazana et al. (2020) to investigate the influence of yolkin on macrophage polarization and morphology, receptor interactions and the expression of specific cytokines.

It was shown that macrophages are critical for the setting up of the immune response against pathogens, maintaining the tissue homeostasis and promoting the repair processes [25]. The upregulation of the innate immune response is regarded as one of the most important strategies to enhance the body’s defense systems, especially in the elderly. There are many observations suggesting that ageing is associated with anti-inflammatory phenotypes, which can diminish the response to different stimulators and the production of insufficient amounts of the pro-inflammatory mediators [10,25]. Several reports have demonstrated a significant effect of some natural active substances such as immunomodulatory protein PEP 1b from *Pleurotus eryngii* [26], peptide complexes such as proline-rich polypeptide complex PRP [27] or plants polysaccharides [28] on macrophage maturation, cytokine expression or RNI or ROS production. In agreement with these reports, our study found that yolkin is bioavailable to the macrophages, significantly stops BMDM cell divisions in about 20–30% of cases compared to the control (Figure 2) and induces morphological changes comparable to those observed in response to LPS (Figure 3). It was found that yolkin labelled with green fluorescent dye CF488A enters the macrophages in a time-dependent manner (Figure 1). It was also shown using HEK-Blue.

^TM^ hTLR2, hTLR4 and NOD2 cells that yolkin is recognized by both TLR2 and TLR4 receptors, but not by NOD2 (Figure 8). On the basis of this data, we can speculate that yolkin binds to and activates TLR 2/TLR4-dependent signaling pathways, triggering iNOS and cytokine expression [7]. Moreover, it can also be assumed that yolkin undergoes endocytosis on its own, or/and in complex with the receptor, as suggested in our previous studies [7].

Macrophages internalize and process antigens to present them to lymphocyte T through MHC-II molecules [29]. However, they need some additional costimulatory molecules to induce a full T cell activation. CD80 and CD86 are specific M1 surface markers that play a significant role in the initial inflammatory response to pathogens [30]. These markers are expressed on the macrophage surface in response to pathogens, which finally leads to the induction of intracellular signaling pathways, such as those controlled by NF-κB, MAPK and PI3K/AKT, and, finally, trigger full T cell activation [31]. We observed that yolkin significantly upregulated CD80/CD86 surface marker co-expression (54.6%) in BMDM cells compared to untreated controls (7.4%). A comparable result was obtained for LPS when it was used as a reference inductor (67%) (Figure 4). These results provide evidence that yolkin is able to activate the M1 macrophage population.

M1 macrophage polarization was paralleled by the secretion of a distinct set of cytokines or chemokines, with high amounts of the proinflammatory Th1-priming cytokines, such as TNFα, IL-6 and also NO, released by LPS-treated macrophages [17,32]. Previously obtained results showed that yolkin treatment significantly upregulated the expression of proinflammatory IFNβ1 and TNFα and also iNOS, which is responsible for the production of nitric oxide [7]. TNFα is a proinflammatory cytokine responsible for the induction and control of the immune response and inflammation [33], while type I IFNs play a critical role in host defense against pathogens, especially in successful viral clearance. However, Karimi et al. [34] showed that type I IFNs are also able to regulate macrophage activity through, e.g., the upregulation of MHC II expression and enhancing its killing activity. Through TLR signaling, macrophages are capable of inducing type I IFN [35]. In turn, iNOS activation and nitric oxide production in activated macrophages play an essential role as a cytotoxic mediator [17,36]. Currently obtained results showed that yolkin stimulated macrophages to increase the level of the proinflammatory IL-6 mRNA (Figure 5b) and also anti-inflammatory IL-10 (Figure 5c) and TGFβ mRNA (Figure 5d). However, it is interesting that IL-10 has long been considered to be an M2 marker, because higher levels of IL-10 were released mainly by M2-polarized macrophages [37,38]. It can, therefore, be concluded that the ability of yolkin to promote the expression of both pro- and anti-inflammatory mediators may suggest its immunoregulatory potential. Yolkin may allow the rearrangement of the M1 into M2 phenotype or generate an intermediate state where pro- and anti-inflammatory mediators are equally important and protect against long-term inflammation.

The upregulation of the mRNA expression of studied cytokines, after treatment with yolkin, was confirmed at the protein level for IL-6 (Figure 6a) and IL-10 (Figure 6b) and for TNFα and type I Interferons, which has been shown previously [7]. Similar studies on innate immune modulators have emphasized the effects of immunomodulatory protein PEP 1b from *Pleurotus eryngii* [26] on macrophage activation and the secretion of IL-6, TNFα and IL-1β. Our results confirmed that yolkin displays significant immunomodulatory activities in BMDM macrophages.

The activation of MAPK and PI3K/Akt kinase pathways is critical in both proinflammatory and anti-inflammatory responses in TLR-stimulated macrophages and has been considered to be a crucial regulator of TLRs and NF-κB signaling in macrophages. In mammals, two major MAPK subgroups are: the extracellular signal-regulated kinases (ERK1/2) and the JNK/stress-activated protein kinases (JNK). ERK1/2 responds to extracellular mitogens and the growth factors that regulate cell proliferation, differentiation and cytokine production [39]. The LPS-dependent activation of JNK kinase was shown to regulate the production of TNFα, IL-1β and IL-6 in murine macrophages [39,40]. Additionally, NF-қB activated by LPS treatment has also been shown to be crucial in the transcription of genes coding IL-6, TNFα and IL-1 in macrophages [41]. In turn, the PI3K/Akt kinases regulate macrophage survival, migration and proliferation, and also orchestrate the response to inflammatory signals in macrophages. The PI3K/Akt pathway, similarly to MAPK, is activated by TLR4 and other pathogen recognition receptors, and can also be activated by cytokines and chemokines, modulating downstream signals that control cytokine production. However, Akt activation is also required for M2 activation and IL-10 production [42]. It was previously demonstrated that yolkin can be a potential TLR4 activator, as it triggers the activation of ERK1/2 and JNK kinases, which subsequently activates the production of inflammatory mediators, such as TNFα, type I interferons and also nitric oxide. Additionally, using the pharmacological inhibitors of MAPK, we revealed that yolkin upregulated the JNK-dependent expression of iNOS, which resulted in the enhanced production of nitric oxide [7]. In the present study, we confirmed that yolkin is recognized by the TLR4 receptor and also very strongly by the TLR2 receptor (Figure 8). Next, we delineated the molecular mechanisms of cytokine induction, by yolkin, in macrophages using pharmacological inhibitors of MAP kinases, namely, U0126 (ERK inhibitor), SP600125 (JNK inhibitor) and LY294002 (PI3K/Akt inhibitor), to suppress their activity. It was observed that the pretreatment of BMDM cells with these three inhibitors (used separately) significantly reduced the yolkin-dependent mRNA expression of proinflammatory TNFα (Figure 7b) and also anti-inflammatory IL-10 (Figure 7e). It was also shown that JNK and PI3K/Akt participated in the regulation of IFNβ1 (Figure 7a) and IL-6 (Figure 7c) mRNA expression; however, the blocking of ERK1/2 kinase activity by the U0126 inhibitor significantly upregulated both IFNβ1 and IL-6 mRNA expression. In the case of TGFβ, the pretreatment of BMDM cells with inhibitors significantly reduced yolkin-dependent TGFβ expression only with MEK1/2 kinase inhibitor-U0126 (Figure 7d).

Our results suggest that both the MAPK and PI3K/Akt signaling pathways are activated in macrophages in response to the yolkin complex and trigger a classical M1 macrophage response, which is comparable with the results obtained by Torres-Martines et al. [43] or Li et al. [44]. It is also noteworthy that yolkin regulates anti-inflammatory IL-10 and TGFβ expression, which may suggest its modulatory, rather than regulatory, action and the ability to control the inflammatory response of macrophages.

## 4. Materials and Methods

### 4.1. Materials

High-glucose Dulbecco’s modified Eagle’s medium (DMEM), phosphate-buffered saline (PBS) (pH 7.4) and trypsin-EDTA were sourced from the Laboratory of General Chemistry of the Institute of Immunology and Experimental Therapy, PAS (Wroclaw, Poland). Tris (hydroxymethyl) aminomethane (Tris), Sephacryl 100-S HR resin, bacterial lipopolysaccharide (LPS) from E. coli (serotype 055:B5), 3-(4,5-dimethylthiazol-2-yl)-2-5-diphenyltetrazolium bromide (MTT) and Tween-20 were purchased from Sigma (St. Louis, MO, USA). L-glutamine and antibiotics (penicillin/streptomycin mixture) were purchased from BioWest (Nuaillé, France). Reagents for SDS-PAGE were purchased from Bio-Rad (Hercules, CA, USA). Molecular weight marker PageRuler™ Plus Prestained Protein Ladder, 10 to 250 kDa was purchased from Thermo-Scientific (Waltham, MA, USA). The U0126 inhibitor was obtained from Cell Signaling Technology (Leiden, The Netherlands), and SP600125 and LY294002 inhibitors were obtained from MedChemExpress (Monmouth Junction, NJ, USA). The antibodies used for flow cytometry analysis were: APC hamster anti-mouse CD80 (clone 16-10A1, BD Pharmingen, San Diego, USA) and BV605 rat anti-mouse CD86 (clone GL1, BD Horizon, Franklin Lakes, NJ, USA).

### 4.2. Cell Lines

The murine bone marrow-derived macrophages of the BMDM cell line (Bei Resources) were maintained in Dulbecco’s modified Eagle’s medium (DMEM) supplemented with 10% FBS, antibiotics (penicillin, streptomycin and gentamycin) and 3% L-glutamine. Cells were grown under standard conditions in a humidified incubator at 37 °C in an atmosphere of 95% air and 5% CO_2_. Adherent cells from confluent cultures were detached, centrifuged at 150× *g* for 5 min and suspended in a complete culture medium.

### 4.3. Isolation of Yolkin Polypeptide Complex

Yolkin was obtained via the standard procedure described in detail by Polanowski et al. [4]. Briefly, the water solution of IgY preparation was the starting material for the isolation of immunologically active peptides. The native IgY, isolated from hen egg yolks after being dialyzed for two days against two changes of 100 mM of potassium phosphate buffer, kept at pH 7.2 and clarified by centrifugation, was chromatographed on a Sephacryl S-100 HR column (K50/100 Pharmacia Ltd., Kent, UK) equilibrated with the same buffer. Fractions, separated from the IgY sample named yolkin, were pooled, dialyzed against water and lyophilized.

### 4.4. MTT Test

Viability and proliferation were determined using MTT colorimetric assay [45]. Briefly, BMDM cells were seeded onto a 96-well plate (1 × 10^4^/well) and incubated overnight in 5% CO_2_/95% air at 37 °C, in a 10% FBS complete medium. The next day, the medium was replaced with a fresh one and the cells were stimulated with yolkin preparation (10–150 μg/mL) or LPS (1 μg/mL), which inhibits macrophage proliferation. After 48 h, the supernatant was removed and the cells were incubated with an MTT reagent (5 mg/mL) for 4 h at 37 °C. Next, 100 µL of DMSO was added onto the plate to dissolve the formed formazan crystals. The absorbance was measured using an EnSpire™ 2300 microplate reader (Perkin Elmer, MA, USA) at 570 nm. Growth was expressed as a percentage of cells versus control cells (100%).

### 4.5. Fluorescent Labelling and Flow Cytometry Procedures

The flow cytometry analysis was performed using LSR Fortessa (Becton Dickinson) units with the following lasers: UV (355 nm), violet (405), blue (488 nm) and red (640 nm), with Diva software. The main population was marked based on the forward and side scatter. The debris was excluded from the analysis by the forward scatter gating. The dead population was removed from the analysis by the DAPI staining. The post-measured analysis was performed using FlowJo VX.07 software (Tree Star Inc, Ashland, OR, USA).

#### 4.5.1. The Uptake of Yolkin by BMDM Cells over Time

BMDM cells were harvested with trypsin-EDTA and their cell membrane was stained with a PKH26 Red Fluorescent Cell Linker Kit (PKH26GL, Sigma-Aldrich), according to the manufacturer’s procedure, seeded on 24-well flat-bottomed plate and cultured overnight in 10% FBS complete medium. The next day, the cells were stimulated with stained yolkin (100 µg/mL) for 15, 30 and 60 min. After stimulation, the cells were harvested with warm PBS, washed twice in 1% FBS in PBS (1300 rpm, 5 min) and stained with DAPI (Sigma) for 10 min at room temperature in the dark. Then, the cells were washed twice, as previously described, and suspended in 400 μL of 1% FBS in PBS for further analysis.

Yolkin was stained with green fluorescent dye CF488A (CF488A succinimidyl ester, Sigma Aldrich, Saint Louis, MO, USA). Briefly, 1 mg of yolkin, reconstituted in 0.1 M sodium bicarbonate buffer (pH 8.3), was mixed with CF488A (1 mg/mL) and incubated for 1 h with agitation, RT, in the dark. Then, the solution was purified using the desalting column (PD MiniTrap-25, GE Healthcare). The concentration of purified and labelled yolkin was determined using the BCA kit (Pierce BCA Protein Assay, Thermo Scientific, Waltham, MA, USA) according to the manufacturer’s procedure. The efficiency of the labelling process was checked by running SDS-PAGE (12% gel) and using a gel imaging system PXi (Syngene, Cambridge, UK).

#### 4.5.2. CD80 and CD86 Expression on BMDM Cells

BMDM cells were seeded on 24-well flat-bottomed plate (3 × 10^5^/well) and cultured overnight in 10% FBS complete medium (37 °C, 5% CO_2_/95% air). Next, the cells were stimulated with yolkin (100 µg/mL) or LPS (1 µg/mL) for 24 h. The next day, cells were stained with DAPI (Sigma) and antibodies for flow cytometry analysis: APC hamster anti-mouse CD80 (clone 16-10A1, BD Pharmingen) and BV605 rat anti-mouse CD86 (clone GL1, BD Horizon). Briefly, the staining procedure was as follows: cultured cells were harvested with warm PBS, washed twice (1300 rpm, 5 min) and stained with DAPI (10 min, RT, in the dark). Then, cells were washed with 1% FBS in PBS (1300 rpm, 5 min), suspended in 20% FBS and incubated for 30 min (4 °C, in the dark) to reduce the non-specific binding of antibodies. The cells were washed twice, as previously described, then suspended in 1% FBS in PBS and labelled with CD80 (1:100) and CD86 (1:100) antibodies (30 min, 4 °C, in the dark). Next, the cells were washed twice and suspended in 400 µL of 1% FBS in PBS and used for analysis.

### 4.6. Morphological Assessment with a Light Microscope

For morphological analysis, BMDM cells were seeded on 6-well plates (3 × 10^4^/mL) and stimulated the next day for 24 and 48 h with yolkin (10 or 100 µg/mL) or LPS (1 µg/mL). The cells were directly observed in their plates and images were taken with a light microscope (Leica DM IRE2, bright field) under phase contrast 4× lenses.

### 4.7. Quantitative Real-Time PCR

BMDM cells (1 × 10^6^/mL) were pre-incubated in the presence or absence of selected kinase inhibitors, 20 μM U0126 (ERK1/2), 25 μM SP600125 (JNK) and 20 μM LY294002 (PI3K/Akt), for 1 h. Next, the cells were stimulated with yolkin (100 μg/mL) or LPS (1 µg/mL) for 4 h. After stimulation, total RNA was isolated from the cells and the expression of iNOS, IFNβ1, IL-6, TNF-α, TGF-β1 and IL-10 were determined via real-time PCR. Total RNA was isolated from the cells using TRI Reagent (Sigma Aldrich), followed by chloroform and isopropanol precipitation, according to the manufacturer’s instructions. The obtained pellet was washed with 75% EtOH and dissolved in RNAse free water. The RNA concentration was measured at 260 nm and at a 260/280 ratio in a BioPhotometer (EppendorfAG, Germany). Next, 1 μg of total RNA was incubated with deoxyribonuclease I (DNase I, RNase-free, Thermo Scientific) to remove trace amounts of genomic DNA. RNA was transcribed to cDNA using M-MLV reverse transcriptase (Promega). cDNA was subjected to qPCR with GoTaq qPCR Master Mix with BRYT Green dye (Promega) on a real-time PCR system (CFX Connect Real–time System, Bio–Rad). For the amplification of the specific genes, the following primers were used: IFNβ1, forward: 5′-AACTTCCAAAACTGAAGACC-3′ and reverse: 5′-AACTCTGTTTTCCTTTGACC-3′; TNF-α, forward: 5′-CTATGTCTCAGCCTCTTCTC-3′ and reverse: 5′-CATTTGGGAACTTCTCATCC-3′; iNOS, forward: 5′-CCGAAGCAAACATCACATTCA-3′ and reverse: 5′-GGTCTAAAGGCTCCGGGCT-3′; IL-10 forward: 5′-CAGGACTTTAAGGGTTACTTG-3′ and reverse: 5′-ATTTTCACAGGGGAGAAATC-3′; IL-6 forward: 5′-GTCTATACCACTTCACAAGTC-3′ and reverse: 5′-TGCATCATCGTTGTTCATAC-3′; TGF-β1 forward: 5′-GGATACCAACTATTGCTTCAG-3′ and reverse: 5′-TGTCCAGGCTCCAAATATAG-3’.

For each mRNA quantification, the housekeeping gene β-actin (*Actb*) was used as a reference gene using the following primers: forward: 5’-GATGTATGAAGGCTTTGGTC-3’ and reverse: 5’-ATTTTCACAGGGGAGAAATC-3’. Real-time PCR data were analyzed using the 2–ΔΔCT method.

### 4.8. Assay for Cytokine Secretion

BMDM cells (1 × 10^6^/mL) were seeded on 24-well flat-bottomed tissue culture plates and cultured overnight in Dulbecco-modified medium. The next day, the cells were treated with yolkin at doses of 10 and 100 µg/mL. LPS (1 µg/mL) was used as a positive control, which activates macrophages to release IL-10. After 24 h of stimulation, the level of cytokines in cell culture supernatants were determined using an ELISA assay with a Mouse IL-10 ELISA Max™ Deluxe Kit (BioLegend, San Diego, CA, USA), a Human/Mouse TGF beta1 Elisa Set (Thermo Fischer Science, Waltham, MA, USA) and a Mouse IL-6 Duo-Set Elisa (R&D System), according to the procedure recommended by the manufacturer.

### 4.9. HEK-Blue^TM^ Cell Cultivation and Stimulation

HEK 293 cells (Invivogen, San Diego, CA, USA) were initially cultured in DMEM with 10% (*v/v*) fetal bovine serum, 100 U/mL penicillin, 100 mg/mL streptomycin and 100 mg/mL Normocin™ (Invivogen, San Diego, CA, USA). After the 3rd passage, selective antibiotics were added to the growth medium as required: (1) null: 100 μg/mL of Zeocin^TM^ (Invivogen, San Diego, USA); (2) TLR2 and TLR4: 1X HEK-Blue^TM^ selection (Invivogen, San Diego, USA); (3) NOD2: 30 μg/mL of blasticidin (Invivogen, San Diego, USA) and 100 μg/mL of Zeocin (Invivogen, San Diego, USA). The cell cultures were renewed with the use of PBS and without centrifuging when confluency reached 80% of the bottle. Finally, the cells were kept at 37 °C with 5% CO_2_ and appropriate humidity.

On the day of the stimulation, cells were detached with PBS, counted and dissolved in HEK-Blue^TM^ Detection (Invivogen, San Diego, CA, USA) (~140,000 cells/mL). Then, 190 μL of the cell suspension was added to each well in the 96-well plate and stimulated with the studied samples (10 µL). Since HEK 293 cells are co-transfected with SEAP (secreted embryonic alkaline phosphatase) genes, stimulation followed by the activation of analyzed receptors gave a colorimetric reaction that was developing over time and which was measured with an absorbance read at 490, 510 and 530 nm.

### 4.10. Data/Statistical Analysis 

Statistical analysis was performed using the software GraphPad Prism 9.1.0 (San Diego, CA, USA). Comparisons were made between groups using a one-sample *t*-test or a one-way ANOVA. The value of *p* ≤ 0.05 was considered to be statistically significant.

## 5. Conclusions

The ageing of the immune system is associated with significant changes in the distribution and competence of immune cells, especially macrophages, leaving the older population more susceptible to infection or cancer and unprotected from chronic tissue inflammation. The possibility of potential therapeutic intervention to inhibit or modulate the ageing processes of the immune system provides tools to control ageing-related diseases and would have a major impact on the health of society.

Our study revealed that the yolkin polypeptide complex, obtained from a natural source, is bioavailable to BMDM macrophages, inhibits its proliferation and upregulates the expression of CD80/CD86 and M1 surface marker expression. It was shown that yolkin promotes the mRNA expression of proinflammatory TNFα, IFN1β, IL-6 and iNOS and also anti-inflammatory cytokines IL-10 and TGFβ. Further, the yolkin-dependent upregulation of cytokine mRNA expression is dependent on MAP and PI3K/Akt kinase activation in BMDM cells. The obtained results clarify the mechanism of action of yolkin and determine the yolkin complex as a potential immunoregulator able to trigger the M1 and M2 phenotype of macrophages.

## Data Availability

All data generated or analyzed during this study are included in this published article.
